# Inference of monopartite networks from bipartite systems with different link types

**DOI:** 10.1038/s41598-023-27744-8

**Published:** 2023-01-19

**Authors:** Kestutis Baltakys

**Affiliations:** Statistical Data Analytics, Faculty of Information Technology and Communication Sciences, Tampere University, Tampere, Finland

**Keywords:** Computational science, Statistics, Complex networks

## Abstract

Many of the real-world data sets can be portrayed as bipartite networks. Since connections between nodes of the same type are lacking, they need to be inferred. The standard way to do this is by converting the bipartite networks to their monopartite projection. However, this simple approach renders an incomplete representation of all the information in the original network. To this end, we propose a new statistical method to identify the most critical links in the bipartite network projection. Our method takes into account the heterogeneity of node connections. Moreover, it can handle situations where links of different types are present. We compare our method against the state-of-the-art and illustrate the findings with synthetic data and empirical examples of investor and political data.

## Introduction

To explain the collective behavior of systems comprised of many interacting parts, it is necessary to understand the structure of the interconnections. This is where network methodologies are particularly appealing^1,2^. Network science provides a domain-independent toolset that can be leveraged to investigate a wide array of complex systems. Successful applications include the studies of social interactions^3–5^, financial markets^6–8^, biological^9,10^, ecological systems^11,12^, information or pathogen transfer pathways^13,14^, or the spread of influence^15–17^.

The ongoing data revolution provides ample amounts of high-resolution data sets^18^ that can be considered from the perspective of networks. However, whereas it is relatively straightforward to analyze the key statistical properties of a given network, the inference of the network structure in itself can be demanding. When direct relationships between agents are not observable, computationally inferred interactions from their behavior contribute additional information for examining a given system. This brings both methodological challenges and opportunities to draw new findings. Unfortunately, in many real-world situations, network inference is an under-determined problem as the number of relationships that can be inferred exceeds the number of independent observations. While recent literature proposes various network inference methods and techniques to establish relationships between agents based on the similarity of their behaviors^19–26^, there is a lack of well-established inference methods particularly tailored to deal with bipartite networks containing different link types.

As the name suggests, bipartite networks portray connections between two different types of nodes. Situations where relationships between two types of entities exist are abundant in many research domains^27,28^. In the biological sciences, bipartite networks represent ecological networks, e.g., plant-pollinator^29^, predator-prey networks^30^, or gene-disease associations^31^. In social sciences, bipartite networks typically represent connections between individuals and some other type of entities. Well-known examples include actors and movies they starred in^32^, individuals and events they attended^33^, authors and papers they contributed to^34^, trade networks^35^, networks of boards of directors^36^, or legislative co-sponsorship networks^37,38^.

The structure of bipartite networks is often leveraged to focus on one set of nodes by creating a one-mode projection, also referred to as a monopartite network. In this paper, we are precisely interested in statistically validated one-mode projections from bipartite networks and propose a new network inference method. The method is tailored for situations where the links in bipartite structures can be of a qualitatively different type. Importantly, the inference procedure considers the heterogeneity of agent activity levels and the heterogeneity and likelihood of different link types. Our method relies on a null model where we exactly fix the degree sequence only for the agent nodes, and we preserve the number of different link types they use as ensemble expectations. The probability of random overlap based on the null model can be evaluated using the hypergeometric-binomial mixture distribution. A statistical test assesses the significance of link similarities in the bipartite network for pairs of agents and determines non-spurious relationships. Two agents are linked if they have a statistically significant number of events to which they are connected via the same type of links. The inferred relationships provide a valuable resource shedding a different light on the observational data and finding novel insights that the data does not contain directly.

In the result section, we use the famous Zachary Karate Club as the ground-truth monopartite network to generate an ensemble of synthetic bipartite networks. We use the synthetic data to demonstrate the performance advantage of our method against an established network inference method in reconstructing the ground-truth network. Moreover, we apply our inference method with two real-world data sets. First, we use parliament member voting data to determine similarities in their decision-making. Second, we use investor trading data to identify statistically significant similarities in investor trading patterns.

## Methods

A bipartite network is defined as $G=(U, V, E)$. Here, *U* and *V* are two disjoint sets of nodes, and *E* is a set of edges $(u, v) \in E \subseteq U \times V$, such that $u\in U$ and $v \in V$. The difference between the bipartite and the monopartite network definitions is that the nodes are composed of two disjoint sets. The links exist only between the two sets of nodes and never between the nodes of the same set. Typically in bipartite data sets, connections between nodes of the same type are not recorded and are unknown. The structure of these bipartite networks is often leveraged to understand the relationships, properties, and function of nodes, typically focusing on one partition of the network. For this reason, such systems are often used to create one-mode projected networks where the connections are established only between nodes of one type^39^. Many data sets with an inherent bipartite system have been used to construct projected one-mode networks, e.g., investor trade synchronization networks^25,40^, asset portfolio overlap networks^41^, or disease networks^42^.

To project a bipartite into a monopartite network where all the nodes belong to the set *U*, we start by linking nodes $i,j \in U$ if in the bipartite system they both share at least a single common neighbor: $\exists k \in V$ such that $(i, k) \in E$ and $(j, k) \in E$. We define the number of neighbors the node $i\in U$ has in the set *V* as $N_i = \sum _{k\in V}\mathbbm {1}_{\{(i, k)\in E\}}$, and the number of shared neighbors by nodes *i* and *j* as $N_{ij} = \sum _{k \in V}\mathbbm {1}_{\{(i, k)\in E\}}\cdot \mathbbm {1}_{\{(j, k)\in E\}}$. The projected network contains links of varying importance in terms of the number of shared neighbors in the bipartite system $N_{ij}$ and the individual activity levels of the connected nodes $N_i$, $N_j$. To separate the strong links from the weak, we need to distinguish which level of connectivity is likely to occur by chance. For example, nodes with many connections are statistically more likely to share more common neighbors than nodes with fewer connections. A straightforward approach to filtering links is to apply an arbitrary global threshold. Nodes with more than the threshold number of neighbors are considered to have a relationship. Nodes co-connected to less than the threshold number of neighbors are considered not to have one. A widely used threshold is zero. It describes a non-filtered projection. If nodes have at least one shared neighbor, they are considered connected. However, such a simplistic approach considers neither the connectivity of individual nodes nor the types of connections.

A more sophisticated way to obtain the monopartite network projection includes using statistical models. The null models are designed by fixing some network properties and randomizing the rest. Nodes are linked only when the number of common neighbors significantly deviates from the one expected under the chosen null model. For example, the bipartite Erdős-Rényi model preserves only the density of the original bipartite network, and the degree sequences in both node sets are random. Under the null model, the number of common neighbors is identical for all pairs of nodes. This choice of the null model resembles an unconditional global threshold.

Alternatively, we can fix the degree sequence for one or both sets of nodes. These null models belong to the family of configuration models. If we restrict the degree sequence only to one of the node sets, we can obtain the probability distribution for common neighbors using the hypergeometric distribution. This approach assumes that the nodes with the non-fixed degree sequence are equivalent and interchangeable. That is, the nodes with the fixed degree sequence are equally likely to connect to any of them. Similarly, we can employ maximum-entropy models where the ensembles preserve the node degrees in either of the sets as ensemble expectations^43^. Filtering a network using a model with both degree sequences fixed requires more sophisticated algorithms^44^.

### A hypergeometric null model

A null model based on the hypergeometric distribution is often used to make statistically validated one-mode projections from bipartite networks^19,20^. We will use it as a state-of-the-art reference null model to compare against our proposed approach. Imagine we are interested in linking nodes *i* and *j*, both of which belong to the same node set *U*, while the set *V* in total has $N=100$ nodes. Let us say *i* and *j* share two neighbors in the bipartite network, i.e., $N_{ij} = 2$. The probability that a similar or larger overlap occurs by chance if *i* randomly chooses three neighbors ($N_i=3$) and *j* randomly chooses four ($N_j=4$) is given by the survival function of the hypergeometric distribution, $H(X \ge 2|100, 3, 4) = 3.58\times 10^{-3}$. Here, the survival function is defined as

1 $$\begin{aligned} H(X \ge N_{ij}| N, N_i, N_j) = 1 - \sum _{X=0}^{N_{ij}-1}H(X| N, N_i, N_j), \end{aligned}$$

where the probability mass function of the hypergeometric distribution is defined as

2 $$\begin{aligned} H(X | N, N_i, N_j) = \frac{{N_i \atopwithdelims ()X}{{N-N_i}\atopwithdelims (){N_j - X}}}{{N\atopwithdelims ()N_j}}. \end{aligned}$$

Alternatively, if both nodes have 30 and 40 neighbors, respectively, the probability of observing a similar or more significant overlap than two common neighbors is $H(X \ge 2|100, 30, 40) > 0.99$. Instead, we could discuss a statistically significant lack of overlap since $H(X\le 2|100, 30, 40) = 2.91\times 10^{-6}$. This example illustrates that the number of common neighbors and the heterogeneity of node connectivity levels play a vital role in distinguishing between the weak and strong links in the projected networks.

### A hypergeometric-binomial mixture null model

Let us consider a similar bipartite system, where links can be of different types. The link types can represent different actions, behaviors, or states through which agents connect to events. The hypergeometric test requires validating different links separately, yielding an ensemble of link-type-specific networks. Suppose the ultimate goal is to investigate the similarity structure of the agents, taking all types of links into account. In that case, the ensemble of networks has to be integrated into a single network. This can be done, e.g., by keeping only the most reoccurring links^45^ or simply taking the union of links over all the networks.

Importantly, each additional link tested increases the chances of making a false positive claim (type I error) about the inferred links. For this reason, link validation is typically concluded with a multiple test correction (MTC). On the one hand, large numbers of tested links increase the probability of false positives. On the other hand, MTC may introduce too strict filtering, discrediting the inference procedure. Although a validated link can suggest strong similarity for specific link types, looking at all link types together, the similarity may be insignificant. This can happen, e.g., due to the similarity of connections in rare link types.

Say agents can use one of the *n* link types ${\textbf{a}} = \{a_1, a_2, \ldots , a_n\}$ to connect to each event. The relationship structure between agents and events is encoded in the incidence matrix ${\textbf{B}}_{|U|\times |V|}$. Here, $|\cdot |$ denotes the cardinality of the set. Elements of the incidence matrix encode the types of links used. If agent *i* is connected to the event *t* via $a_k$ link type, $B_{it} = a_k$. If agent *i* is not connected to event *t*, $B_{it} = 0$. We can retrieve the number of events agent *i* is connected to from the incidence matrix by $\sum _{t=1}^N \mathbbm {1}_{\{B_{it}\in {\textbf{a}}\}} = N_i$, where $\mathbbm {1}$ is an indicator function and $N=|V|$. The number of events to which both agents *i* and *j* are connected is defined as $N_{ij} = \sum _{t=1}^N\mathbbm {1}_{\{B_{it}\in {\textbf{a}}\}}\cdot \mathbbm {1}_{\{B_{jt}\in {\textbf{a}}\}}$. The number of events to which both agents are connected via the same link types is defined as ${\tilde{N}}_{ij} = \sum _{t=1}^N\mathbbm {1}_{\{B_{it} = B_{jt}\}}$. In general, ${\tilde{N}}_{ij}$ and $N_{ij}$ range as $\max (N_i + N_j - N, 0) \le {\tilde{N}}_{ij} \le N_{ij} \le \min (N_i, N_j)$.

Additionally, we need to consider the agents’ link type preferences. For this we empirically estimate ${\textbf{p}}_i = \{p_i(a_1), \ldots , p_i(a_n)\}$, where $p_i(a_k) = \sum _{t=1}^N\mathbbm {1}_{\{B_{it} =a_k\}}/N_i$. Alternatively, link-type preferences could be estimated over the whole population of agents, i.e., $p(a_k) = \sum _{i,t}\mathbbm {1}_{\{B_{it} =a_k\}}/\sum _{i}N_i$. The probability that two agents randomly use the same link type in a given event is defined as $p_{ij} = \sum _{k=1}^n p_i(a_k)p_j(a_k)$. Agents choose different link types with probability $1 - p_{ij}$.

How significant is the observed number of shared events with the same link types ${\tilde{N}}_{ij}$, given the total number of events *N*, individual agent activity levels $N_i$, $N_j$, and agent preferences for different link types ${\textbf{p}}_i$, ${\textbf{p}}_j$? Under the null model, agents randomly choose events to connect to and link types. The hypergeometric distribution $H(N_{ij} | N, N_i, N_j)$ defines the probability for agents *i* and *j* to choose the same $N_{ij}$ events to connect to by chance. The probability that both agents choose the same link types ${\tilde{N}}_{ij}$ times, over the $N_{ij}$ events they are jointly connected to, follows the binomial distribution defined as

3 $$\begin{aligned} B(Y = {\tilde{N}}_{ij} | N_{ij}, p_{ij}) = {N_{ij} \atopwithdelims (){\tilde{N}}_{ij}} p_{ij}^{{\tilde{N}}_{ij}}(1-p_{ij})^{N_{ij} - {\tilde{N}}_{ij}} \end{aligned}$$

To evaluate how significant is the observed overlap ${\tilde{N}}_{ij}$, we need to evaluate the probability of seeing the same or a more extreme value by chance. This probability can be evaluated using the hypergeometric-binomial mixture distribution defined as

4 $$\begin{aligned} {\mathbb {P}}(Y \ge {\tilde{N}}_{ij}| N, N_i, N_j, p_{ij})= & {} \sum _{X = {\tilde{N}}_{ij}}^{\min (N_i, N_j)} H(X | N, N_i, N_j) B(Y \ge {\tilde{N}}_{ij}|X, p_{ij}) \nonumber \\= & {} \sum _{X = {\tilde{N}}_{ij}}^{\min (N_i, N_j)} H(X| N, N_i, N_j) \sum _{Y = {\tilde{N}}_{ij}}^{X} B(Y|X, p_{ij}). \end{aligned}$$

Eq. 4 does not include the number of events $N_{ij}$ that both agents connect to. The key is that both agents use the same link types to connect to ${\tilde{N}}_{ij}$ or more events. While the proposed null model takes into account the degree distribution in the node set *U*, like the hypergeometric null model, it assumes nodes of the set *V* to be interchangeable, i.e., having the same probability of being connected to by nodes in set *U*. In some situations, this assumption is unjustifiable. However, if the setting of the investigated complex system does not imply that the nodes in the set *V* are somehow unique, the assumption does not cause a significant issue. Alternatively, as proposed by Tumminello et al.^19^, one could perform separate link validation for multiple batches of nodes in set *V* according to their connectivity levels.

**Figure 1:**
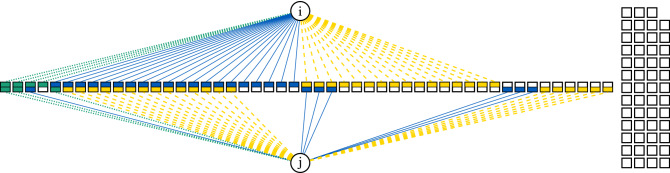
An illustration of a bipartite network focusing on two agents *i* and *j* represented as  and their connections to 100 different events represented as . Each agent connects to events via three different types of links (, , ). Additionally, the squares representing events are divided into two halves. The color of the top half indicates the type of connection node *i* has to this event, while the color of the lower half indicates the connection type of node *j* ($a_1 =$ , $a_2=$ , $a_3=$ ). Unfilled event squares indicate a lack of connection from nodes *i* and/or *j*.

Let us take an example with two agents, *i* and *j*, and 100 events. Let us say that each time an agent connects to an event, he can choose from three distinct link types ${\textbf{a}} = (a_1, a_2, a_3)$. An example contingency table for the links used by two agents is shown in Table 1. The corresponding bipartite network is illustrated in Fig. 1.

**Table 1:** Contingency table summarizing the connections of agents *i* and *j* over 100 events, also illustrated in Fig. 1.

*j*	*i*				
	$a_{1}$	$a_{2}$	$a_{3}$	–	$N_{j}^{a}$
$a_{1}$	2	1	0	0	3
$a_{2}$	1	0	3	3	7
$a_{3}$	0	14	0	6	20
–	1	5	13	51	70
$N_{j}^{a}$	4	20	16	60	100

When considering the overlap in link type $a_1$, agent *i* chose it four times, agent *j* chose it three times, and together they chose it two times. From the earlier example, we know that this is a statistically significant link overlap with a *p* value of $3.58\times 10^{-3}$. Even though there is no overlap in other link types, aggregating the three link-specific networks would yield a connection between these two agents. Such a conclusion is biased, leading to the overlap in rare events to dominate over the lack of overlap in more frequent events. If we were to infer the significance of overlap over all different types of links, intuitively, we would expect a different result.

For each agent we estimate unconditional probabilities to choose a certain link type. From the Table 1 we have that ${\textbf{p}}_i = (0.1, 0.5, 0.4)$ and ${\textbf{p}}_j = (0.1, 7/30, 20/30)$. Therefore, the probability that agents choose the same link type given that they both choose to connect to the same event is $p_{ij} = 118/300 = 0.39(3)$. The probability that *i* and *j* choose the same link type for two or more events together is given by Eq. 4, i.e., ${\mathbb {P}}(Y \ge 2| N=100, N_i=40, N_j=30, p_c=0.39(3)) \approx 0.96$. Taking all of the link types together we cannot conclude that the connections of agents *i* and *j* exhibit a statistically significant overlap. At the same time we can observe that out of $N_{ij} = 21$ shared events, in $N_{ij} - {\tilde{N}}_{ij} = 19$ they have used different link types. The probability of such a lack of link overlap under the null model is highly unlikely, i.e. ${\mathbb {P}}(Y \ge 19| N, N_i, N_j, 1 - p_{ij})) = 6.48 \times 10^{-7}$.

### Signed networks

**Figure 2:**
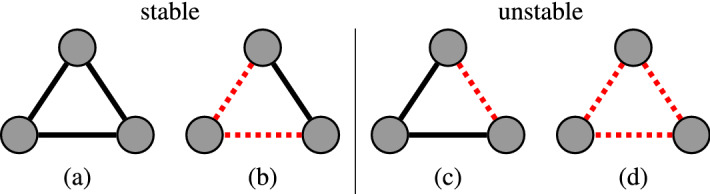
According to the structural balance theory, (**a**,**b**) are stable triadic motifs, while (**c**,**d**) are unstable. Solid black lines () indicate positive links, while the negative links are indicated by dotted red lines ().

We can leverage the introduced hypergeometric-binomial null model to statistically validate positive and negative links, thus yielding a signed network projection. Signed networks can be studied from the perspectives of balanced relationships^46,47^. For example, three connected nodes in a signed network can be categorized as either having a stable or an unstable triadic relationship. The categorization is intuitive from the perspective of social networks. *Stable triads* either have three positive relationships (a friend of a friend is my friend, see Fig. 2a), or two negative relationships and one positive (an enemy of my friend is my enemy, see Fig. 2b). *Unstable triads* either have one negative and two positive (see Fig. 2c), or three negative relationships (see Fig. 2d). In the unstable triads, all of the relationships are subject to change. When all the relationships are negative (Fig. 2d), each can be tempted to switch from a negative relationship to a positive one, thereby creating an alliance against the remaining node. When there is one negative and two positive relationships (Fig. 2c), the nodes sharing a negative tie are incentivized to switch to a positive relationship because they share a common “friend”. Alternatively, one of the positive relationships can switch to a negative.

We can obtain positive links from statistically significant overlap in the same link types. Similarly, negative links can be obtained from a statistically significant number of events where agents choose opposing link types. In some settings, certain link types can be considered to oppose each other. As illustrated in the result section, investors can have a buy or sell connection to securities, and politicians can cast a supporting or opposing vote.

To infer a negative link between agents, we need to count the number of shared events where they have used opposing links $N_{ij}^d$. Moreover, we need to estimate the probability that agents oppose each other by chance,

5 $$\begin{aligned} q_{ij} = \sum _{(a_+, a_-)} (p_i(a_+)p_j(a_-)+p_i(a_-)p_j(a_+)), \end{aligned}$$

where $(a_+, a_-)$ are pairs of link types that can be considered as opposing. The statistical significance of overlap in opposing link types is evaluated via ${\mathbb {P}}(Y \ge N^d_{ij}| N, N_i, N_j, q_{ij})$. Continuing our example, we consider $a_1$ a neutral link type, and $a_2$ and $a_3$ opposing link types. The probability for agents *i* and *j* to choose opposing link types by chance is $q_{ij} = p_i(a_2)p_j(a_3) + p_i(a_3)p_j(a_2)=32/75=0.42(6)$. We have $N^d_{ij}=17$ events to which agents are connected via opposing link types $a_2$ and $a_3$. Therefore the probability of such opposition occurring by chance under the null models is ${\mathbb {P}}(Y \ge 17| N, N_i, N_j, q_{ij})) = 1.70\times 10^{-7}$. The probability that agents *i* and *j* choose so many opposing links by chance is highly unlikely. Therefore we can conclude that the two agents oppose each other in a statistically significant way. In the result section, we investigate how many triadic relationships can be observed in the inferred signed networks and check how many of them are stable.

## Results

In this section, we apply the introduced approach for link validation with the Hypergeometric-Binomial null model with synthetic data and two real-world data sets resulting from human behavior. We use the synthetic data to evaluate the performance of our proposed link validation approach and compare it against a state-of-the-art reference method based on the Hypergeometric null model^19,20^. First, we investigate the differences in the validation of a single link. Next, we leverage a simple influence model on a well-known social network to generate a series of synthetic bipartite networks. The generated data is used to reconstruct the original social network and evaluate the performance of the proposed method. The two real-world data sets illustrate possible applications of our link validation approach. The first real-world data set contains the votes of parliament members in Lithuania. Parliament members (agents) can either support, oppose, or abstain (link types) from deciding when voting on a specific question (events). The second real-world data set contains investor-level trading data from Helsinki Stock Exchange. Investors (agents) may increase or decrease their position or day-trade (link types) on a given trading day (events).

### Improvement in a single link detection

First, we consider a minimal synthetic bipartite network with two agents, 20 events, and three different link types. To illustrate the differences between our proposed and the reference method in single link validation, we create an ensemble of networks with all possible configurations of the bipartite network. We can observe diverse connectivity patterns over the whole spectrum of bipartite networks. Agent’s link type preferences range from equally likely to concentrated in only one of the link types. The overall number of agent connections ranges from just one connection to 20, $1 \le N_i, N_j \le 20$. For each configuration of the bipartite network, we calculate the significance of agents’ link overlaps using our proposed approach with Eq. (4).

We present grouped results based on the networks’ configuration properties: fraction of events with overlapping links, ${\tilde{N}}_{ij}/N$, and concentration of overlap in terms of link types. We measure the concentration with the Herfindahl-Hirschman index:

6 $$\begin{aligned} HH = {\sum _{n=1}^{3}N_{ij}(a_n)^2}/{{\tilde{N}}_{ij}^2}, \end{aligned}$$

where we denote $N_{ij}(a_n)$ as the number of events to which both agents are connected via link type $a_n$.

**Figure 3:**
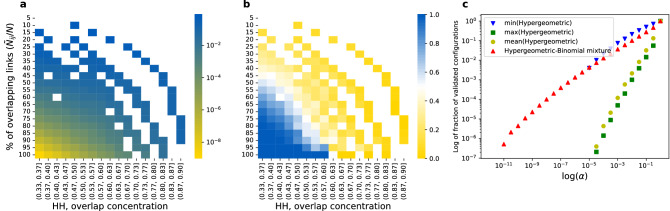
Validation of a single link in a bipartite network with two agents, 20 events, and three different link types. The horizontal axis in (**a**,**b**) shows the concentration of overlap in terms of link types measured with the Herfindahl–Hirschman index, see Eq. (6). The vertical axis in (**a**,**b**) indicates the fraction of events to which both agents are connected via the same link types (${\tilde{N}}_{ij}/N$). (**a**) depicts the mean *p* values of our proposed link validation method for all possible configurations of the bipartite network. (**b**) depicts the fraction of configurations where *p* values provided by our method are lower than the smallest *p* value provided by the reference method over the three link types. Notably, the Hypergeometric-Binomial null model nearly always yields a lower *p* value if compared with the mean or the maximum *p* value provided by the reference method over the three link types, see Fig. S1. (**c**) illustrates the fraction of validated bipartite network configurations with different methods for a given statistical significance level $\alpha$. Both axes are in log scale.

Figure 3a illustrates the average *p* value for a bipartite network configuration group given the Hypergeometric-Binomial null model. As expected, the higher the link overlap, the lower the *p* value suggested by our method. Similarly, the less concentrated the overlap in link types, the lower the *p* value suggested by our method. In other words, our method is more likely to identify the overlap as spurious if it is overall lower and more concentrated in link types.

To compare our proposed approach against a reference, we calculate the significance of different link overlaps using Eq. (1). Figure 3b illustrates the fraction of bipartite network configurations where the Hypergeometric-Binomial null model yields a lower *p* value than the lowest *p* value obtained over all link types with the Hypergeometric null model. On average, our approach yields a lower *p* value, the less concentrated the overlap in terms of link types and the higher the total link overlap. In other words, the reference method is more likely to render a lower *p* value when the number of overlapping links is lower and the overlap is more concentrated in terms of link types. Figure S1 illustrates the comparison against average and maximum *p* values obtained over all links with the reference method. Nearly for all network configurations our method yields lower *p* values.

Finally, Fig. 3c illustrates the fraction of validated bipartite network configurations with different methods for a given statistical significance level $\alpha$. Over the whole spectrum of bipartite networks our proposed method yields a wider range of *p* values. The smallest *p* value obtained using our method is of the order $10^{-12}$ while the smallest observed with the reference method is only $10^{-8}$. Using the minimum *p* value obtained over all link types results in a higher number of validated bipartite network configurations than using the *p* values obtained using our method. Naturally, our method yields a significantly higher number of validated links if we take the average of the maximum *p* value of the reference method.

### Reconstruction of a ground-truth network

In this section, we evaluate the performance of our method in reconstructing a ground-truth monopartite network from bipartite data. First, with a simple influence model, we generate an ensemble of bipartite networks. In the bipartite networks, nodes of the ground-truth monopartite network connect to a set of newly generated nodes via three link types. Second, each bipartite network is projected back to a monopartite network with ground-truth network nodes. Third, the projections are validated using our proposed and reference null model. Finally, the validated networks are evaluated against the ground-truth network, and we compare the performance of our proposed approach against the reference. The performance is measured via four metrics defined in Table S1 in the Appendix: accuracy, precision, recall, and F1.

We use th﻿e Zachary Karate club social network^9^ as the monopartite ground-truth network. The social network is composed of 34 nodes (agents) and 78 social connections between them. To generate the bipartite data, we need neighboring agents to be more likely to connect to the same events. For this, we design a custom influence model. We take inspiration from existing models that have been developed to study the synchronization of agent behavior in social networks^48–50^. These models typically aim to understand how the behavior of individual agents within a group or community can become synchronized or coordinated over time and to identify the factors that influence the emergence of synchronized behavior. Moreover, our influence model most closely resembles the independent cascade model^51^, which can be used to describe the spread of behaviors or ideas within a social network. In the independent cascade model, an individual’s adoption of a behavior or idea is influenced only by their direct connections to other individuals who have already adopted the behavior or idea. The adoption of the behavior or idea then “cascades” through the network as it is passed from one individual to another.

In the influence model, each agent *i* has three attributes: (i) the probability of connecting to an event $n_i \in [0, 1]$, (ii) the probability to mimick neighbor behavior $c_i \in [0, 1]$, and (iii) link type preferences $p_i(a_k) \in [0, 1] : \sum _{a_k}p_i(a_k) = 1$. These attributes are fixed for a given realization of a bipartite network. The first two probabilities are drawn from a uniform distribution, while the link preferences are randomly chosen and can range from a complete concentration on a single link type to an equal preference for all three link types. To generate the bipartite network, event nodes are added one at a time, causing an independent cascade. For each event node, the process starts with a random agent node connecting to it. Then, in the spirit of breath-first-search, the neighbors of the already-connected agent nodes in the ground-truth network decide whether they will connect to the new event node, if so, which link type to use. They can either mimic one of the neighboring agents’ link type or randomly pick according to it’s link preferences. This process is repeated 100 times, resulting in a single bipartite network with 34 nodes from the ground-truth network (representing agents) and 100 newly added event nodes connected via three link types. A more detailed description of the influence model can be found in the Appendix.

The generated bipartite networks are projected back to monopartite networks with ground-truth nodes. We do this by connecting pairs of agents connected to at least one event via the same link type. In each projected network, link overlaps are assessed by assigning a *p* value. We compare the *p* values of our proposed null model against the *p* values obtained with the reference null model over the three link types. In particular, we estimate the AUC metric. It measures how well the *p* values distinguish between the existing and non-existing links in the ground-truth network. Averaged over an ensemble of 1000 simulated bipartite networks, the AUC measure for the *p* values obtained with the Hypergeometric-Binomial null model is 0.78. If we use the minimum *p* value of the reference null model obtained over the three link types, the AUC score is 0.76; if we use the mean, the AUC score is 0.75; and if we use the maximum, the AUC score is 0.64. Using the smallest *p* value of the reference null model obtained over the three link types yields the highest AUC score. We perform a paired two-sample t-test with the null hypothesis that the means of AUCs obtained with our and the best reference model are the same. With a *p* value of $4.33\cdot 10^{-21}$, we reject the null in favor of the alternative: the mean of the AUC score obtained for *p* values resulting from our model is greater than the one obtained from the *p* values of the reference model. Since taking the smallest *p* value of the reference null model over all the link types yields the best AUC, we will use this approach as the main reference to have the toughest benchmark.

Next, we investigate the performance of network reconstruction using different statistical significance levels $\alpha$. Table 2 summarizes the performance of ground-truth network reconstruction obtained over 1000 simulations. F1 score ranges from 47.6% to 5.7% depending on the level of statistical significance $\alpha$. Importantly, we perform a paired two-sample mean t-test and reject the null hypothesis about equal means in favor of the alternative: the mean F1 score resulting from our method is greater for all values of $\alpha$. We find that the more strict the statistical significance threshold, the higher the precision and the lower the recall and the F1 score. That is, on the one hand, a more significant fraction of the validated links will represent the links in the ground-truth network, while on the other hand, fewer of the ground-truth network links will be validated. When $\alpha \le 10^{-4}$, the network reconstruction precision using the reference model is statistically higher than using our proposed model. However, the precision using our proposed method is still higher than 90%. For less stringent statistical significance ($\alpha > 10^{-4}$), the precision of network reconstruction using our model, on average, is statistically higher than using the reference model. Similarly, for most of the significance thresholds $\alpha$, the recall metric is, on average, statistically higher when our proposed network reconstruction method is used. Moreover, our method’s performance in terms of accuracy, recall, and F1 scores is significantly better than the reference if we take the maximum or the mean *p* value over all link types (see Table S3).

**Table 2:** The relationship between the network reconstruction performance measures and statistical link validation threshold $\alpha$.

	Precision			Recall			F1			Accuracy		
$\alpha$	$\langle \text {pr}\rangle$	$\langle \Delta \text {pr}\rangle$	$\ge$	$\langle \text {re}\rangle$	$\langle \Delta \text {re}\rangle$	$\ge$	$\langle \text {F1}\rangle$	$\langle \Delta \text {F1}\rangle$	$\ge$	$\langle \text {acc}\rangle$	$\langle \Delta \text {acc}\rangle$	$\ge$
$$10^{-10}$$	98.0%	$$-\,1.8\%^{**}$$	93%	1.3%	$$1.0\%^{***}$$	99%	5.7%	$$2.8\%^{***}$$	98%	86.3%	$$0.1\%^{***}$$	99%
$$10^{-9}$$	97.9%	$$-\,1.5\%^{*}$$	92%	1.7%	$$1.3\%^{***}$$	98%	6.5%	$$3.4\%^{***}$$	98%	86.3%	$$0.2\%^{***}$$	98%
$$10^{-8}$$	97.7%	$$-\,1.5\%^{**}$$	90%	2.4%	$$1.7\%^{***}$$	98%	7.5%	$$4.0\%^{***}$$	98%	86.4%	$$0.2\%^{***}$$	97%
$$10^{-7}$$	96.9%	$$-\,1.8\%^{***}$$	87%	3.4%	$$2.1\%^{***}$$	97%	8.7%	$$4.5\%^{***}$$	97%	86.5%	$$0.3\%^{***}$$	96%
$$10^{-6}$$	96.2%	$$-\,1.8\%^{***}$$	84%	4.9%	$$2.6\%^{***}$$	96%	10.6%	$$5.0\%^{***}$$	95%	86.7%	$$0.3\%^{***}$$	95%
$$10^{-5}$$	94.6%	$$-\,1.0\%^{*}$$	78%	7.1%	$$3.1\%^{***}$$	95%	13.6%	$$5.3\%^{***}$$	93%	87.0%	$$0.4\%^{***}$$	93%
$$10^{-4}$$	91.8%	$$-\,0.1\%$$	71%	10.7%	$$3.3\%^{***}$$	91%	18.7%	$$5.2\%^{***}$$	89%	87.4%	$$0.4\%^{***}$$	87%
$$10^{-3}$$	86.3%	$$2.7\%^{***}$$	67%	16.7%	$$3.0\%^{***}$$	85%	27.0%	$$4.1\%^{***}$$	81%	87.9%	$$0.4\%^{***}$$	81%
$$10^{-2}$$	73.6%	$$8.5\%^{***}$$	86%	27.8%	$$0.3\%$$	59%	38.9%	$$1.4\%^{***}$$	63%	88.3%	$$0.7\%^{***}$$	85%
$$10^{-1}$$	46.5%	$$10.9\%^{***}$$	100%	50.6%	$$-\,6.5\%^{***}$$	9%	47.6%	$$4.2\%^{***}$$	88%	84.6%	$$5.3\%^{***}$$	100%

The columns with $\langle \cdot \rangle$ indicate the averaged measures obtained with our method. The columns with $\langle \Delta \cdot \rangle$ indicate the mean difference between the measures obtained with our and reference null models. Here the stars indicate the *p* values for a paired two-sample mean t-test, with an alternative that the mean difference of the samples is not zero. Finally, the $\ge$ columns indicate the fraction of simulations where the measures obtained with our null model yielded a similar or better performance compared to the reference model. All results are obtained over 1000 simulations. ***$p < 0.001$; **$p < 0.01$; *$p < 0.1$.

To understand how the network reconstruction performance is affected by the different parameters of the influence model, we simulate different ensembles where one of the parameters $c_i$, $n_i$, or $p_i$ is restricted to some range. We find that the more heterogeneous the link type preferences, the better our model performs in terms of recall and F1 scores. Moreover, the more heterogeneous link type preferences, the more our method outperforms the reference in terms of AUC, recall, and F1 scores (see Panel A in Table S2). In terms of the node activity parameter $n_i$, networks reconstructed using our approach outperform across all performance measures except when the activity parameter is very low or very high (see Panel B in Table S2). Our model slightly underperforms in terms of recall measure compared to the reference for low values of copying parameter $c_i$. However, it significantly outperforms the reference in the mid-range in terms of precision and mid-to-high range in terms of the F1 score.

Overall, our proposed method significantly outperforms the reference method when the link preferences are more heterogeneous, the agent activity level is neither too low nor too high, and agents do not have a low probability of copying their neighbors’ behavior. In other situations, our method is either on par with the reference method or the probability of achieving a similar or higher performance score is more than 50% in most cases.

### Political network

Political networks in recent years have gained more attention from researchers^52^. There are a few typical ways to infer networks from political data. The first one is based on linking individuals that co-sponsor the same legislation^21,37^. The second one is based on linking individuals who vote similarly^53–55^. With the latter link inference approach, a binary projection can be used to link individuals if they have voted the same way at least once. Links can also be assigned weights, measuring the fraction of cases when individuals’ agreed or disagreed during a vote. Those links can then be filtered using a simple threshold^56^ or via k-means^57^. Alternatively, a disparity filter can be used to prune the weighted networks^58^. In this section, we show that our proposed link validation method perfectly fits the problem of political network inference from the voting data. Indeed, our link validation procedure is akin to finding statistically significant similarities or differences between individuals’ voting decisions. The resulting network structure displays parliament members’ ideological similarities and differences.

We use a publicly available data set from the Lithuanian parliament, known as the Seimas of Lithuania. Lithuania is one of the European Union members that operates a multi-party system. The data set is available from the parliament’s official website^59^ and covers a five-term period from 1996 to 2021. It contains information about elected parliament members (agents), their political party affiliations, all voting events, individual parliament member votes (link types) in each event, and the outcomes of those events from 1996 to 2021.

The Lithuanian parliament is composed of 141 members elected for a four-year term. After the election, parliament members negotiate to form the ruling coalition. Non-invited political groups form the opposition. Each term is divided into nine regular sessions, each of which can have an irregular complement. The Lithuanian parliament meets at least twice a year in regular spring and autumn sessions. Parliament members consider, adopt, issue legislation, approve, supervise the state budget, set taxation, and discuss other important issues. Each parliament member can either support, abstain or oppose when voting. Often, members do not participate in those events at all.

It is interesting to see if the political network inferred leveraging our proposed null model is indicative of the political realities. Is there a clear division in the structure between members of the ruling majority and the opposition? Are there more intra- than inter-party links?

A validated parliament member network projection may reveal similar political leanings independent from their political party affiliations. Moreover, occasionally parliament members change their political affiliations and may move from the opposition to the ruling majority or vice versa. Later, we will take a quick look at these events. It is interesting whether the changes in political affiliations can be anticipated from the changes in the statistical significance of their voting similarities.

Leveraging the introduced link validation approach, we infer a signed political network. In this network, members of the parliament are connected via positive links if we observe a statistically significant overlap in their votes. Negative links are established between members that systemically vote in an opposite direction, thereby capturing political antagonisms^21^.

First, we investigate the voting data of the eighth parliament (2000-2004). The election of the parliament members took place on October 8th, 2000. 138 members of 16 political parties were elected, with three members not belonging to any party. After the election, seven parliamentary groups formed – Social Democratic Coalition (SDKF, 48 seats), Liberals (LF, 33 seats), New Union (NSF, 29 seats), Homeland Union - Conservatives (TSLK, nine seats), the Centre Union, the Modern Christian Democrats and the Electoral Action of Poles in Lithuania (JF, eight seats), Peasant and New Democracy Parties (VNDF, seven seats), and the Mixed Group of Members of the Seimas (MG, seven seats). Three parliament groups, LF, NSF, and JF, formed the ruling coalition that lasted less than a year until June 2001.

We infer the political network using the data from the first regular session from 24th October to 23th December 2000. During this session, there were 415 events where parliament members cast their votes. We have further reduced the data set by considering only events where at least 71 parliament members were present. This reduced the number of events to $N=327$. A parliament member can either support, oppose or abstain from deciding (three link types) in a given event. That is, the set of possible link types each member can choose from is defined as ${\textbf{a}}=\{S, O, A\}$.

**Figure 4:**
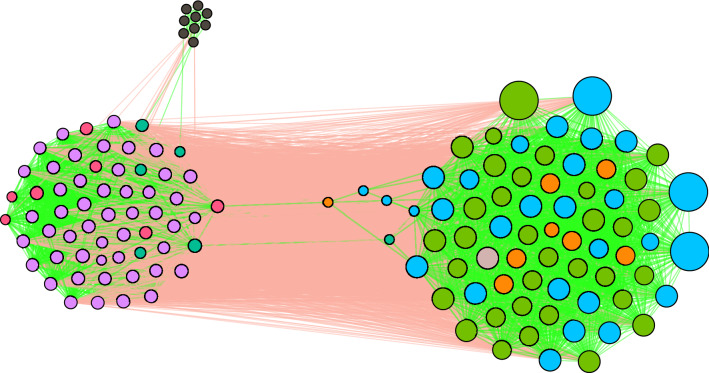
A validated political network inferred from a bipartite network where parliament members vote on different questions. The cluster on the right is composed of members that formed the ruling coalition, while the members on the right were in opposition. The layout is based on the positive (green) links only. The size of the nodes is positively associated to their positive degrees. Node colors represent the political groups in the parliament: —SDKF, —LF, —NSF, —TSLK, —JF, —VNDF, —MG. The grey node in the ruling coalition cluster is the elected speaker of the parliament which belonged to the NSF group.

For each parliament member *i*, we calculate the number of events when he or she was active $N_i$ and the empirical frequency of different voting decisions $p_i(a_k)$, where $a_k \in \{S, O, A\}$. Then, for all pairs of parliament members (*i*, *j*), we calculate the number of events when they both have cast the same (${\tilde{N}}_{ij}$) and opposing ($N^d_{ij}$) votes. Similarly, we estimate the probabilities of doing this by chance $p_{ij}$ and $q_{ij}$. Here we consider the vote abstain *A* as neutral. Naturally, the supporting vote *S* is the opposite of the opposing vote *O*. Having calculated these parameters, we retrieve the corresponding *p* values. Pairs of parliament members with sufficiently low *p* values are linked together in the signed political network, see Fig. 4. Here we also use the Bonferroni multiple test correction with a statistical threshold of $\alpha =0.01$.

Node colors indicate political affiliations. The node size is proportional to the number of positive links a node has. Positive links are indicated with green lines and negative ones with red. The network consists of 140 parliament members (one member of the opposition had neither positive nor negative links). The validation procedure resulted in 3889 positive links and 2232 negative links. Note that the network layout is based only on positive connections, clearly showing the separation of the parliament members into the ruling coalition and the opposition. Positive links are primarily observed between the members of the ruling coalition or between the opposition members. A group of four members from the ruling coalition is an exception. They have few positive links with the other members of the ruling coalition. Moreover, each has at least a single positive link to opposition members. Negative links, on the contrary, are primarily observed between the members of the ruling coalition and the opposition. All nine opposition members with the TSLK affiliation form a connected clique with only positive links. However, they have just three positive and 11 negative links to other members in opposition. Interestingly, the signed political network is very stable in terms of balanced relationships introduced in Fig. 2. We find a total of 171 677 triadic relationships (triangles), and only 1 is unstable.

#### Investigation of changes in political affiliation

The parliament members’ political affiliations are not fixed. There are 238 cases when a politician has changed political affiliations over the five office terms. We leverage the proposed null model to investigate the dynamics of behavioral similarities between the switching politician and the new and old political groups.

First, we choose an observation window of 450 voting events before and after switching the affiliation. The politician must be associated with the old (new) affiliation for at least 450 events before (after) the switching event. We also limit the affiliation-switching cases to those where the members of the new and old affiliations are sufficiently distinctive. We exclude the cases where the Jaccard similarity of the members of the new and old affiliation is more than 0.5. Moreover, we limit to those cases where at least $50\%$ of members affiliated with the old (new) political group were not affiliated with the new (old) political group. With the help of these filters, we exclude the cases where political parties perform re-branding, maintaining the same core of politicians. 46 switching events pass the defined criteria.

We choose a moving window of 120 voting events with a displacement of 30 events. We perform a bipartite network projection within each window and evaluate the statistical significance of vote overlaps using our proposed method. We are interested in the similarity of voting behavior between parliament members who switch their affiliations and parliament members in the old and new affiliations. We measure the similarity with the *p* values resulting from our link validation approach. The higher the *p* value, the higher the dissimilarity. The lower the *p* value, the higher the similarity.

**Figure 5:**
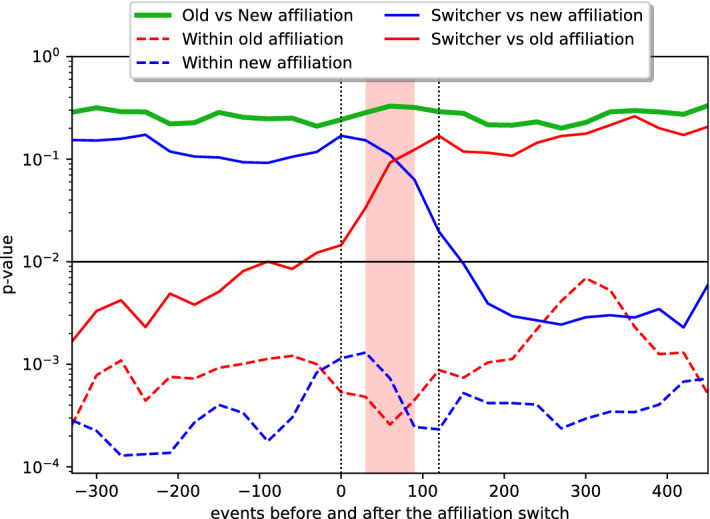
The statistical significance of parliament member voting similarities around affiliation-switching events. The highlighted area in the middle indicates the span where we cannot reject the null hypothesis about equal means with 0.01 statistical significance.

Blue-colored curves in Fig. 5 indicate the similarities related to parliament members in the new affiliation. The dashed line indicates the similarity of parliament members who were part of the new affiliation throughout the whole period around the switching event, which happens at time 0. We see that the median similarity is high, with *p* values significantly below the statistical threshold of 0.01. This means that a median pair of parliament members in the new affiliation would be connected as a consequence of our proposed approach. The solid blue line shows the similarity between the parliament member who switches affiliation and members in the new affiliation. While the similarity is relatively low prior to the switching event, the median of *p* values starts to go down immediately after. Just after the 120th event, past the switching event, when there is no more data about voting prior to the switching, the median *p* value drops below 0.01. However, it does not quite reach the median levels between existing members in the new affiliation (blue dashed curve). Nevertheless, the drop is sufficient to establish a link between the switcher and a median member in the new affiliation.

The similarity towards the old affiliation is indicated with red curves. The red dashed curve indicates relatively high similarity between members in the old affiliation. The solid red line indicates an increasing dissimilarity between the switching member and members of the old affiliation. From the beginning of the window, we can see that the dissimilarity is higher between the switching member and the other members of the old affiliation than between other pairs of members in the old affiliation. Interestingly, the solid red line breaches the 0.01 statistical threshold prior to the switching event. This measure can serve as an early warning signal for brewing changes in the political system. The thick green line indicates low similarity between members in the switchers’ old and new affiliations. We see that the switcher was never as dissimilar to his new colleagues as his old affiliations (blue and green solid curves), nor did he become as dissimilar to his old colleagues as his new colleagues (red and green solid curves).

#### Differences in validated political network

**Figure 6:**
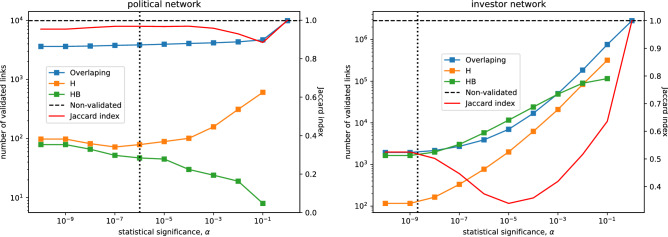
Comparison of empirical networks inferred leveraging our proposed (HB) and the reference (H) null model where the smallest *p* value obtained over all link types is used when validating a link. The horizontal axis indicates different statistical thresholds used in link validation. The left-hand side figure shows the comparison of the political networks. The right-hand side figure shows the comparison of the investor networks.

To understand what difference our proposed approach yields in empirical network inference, we compare the number of inferred links using the Hypergeometric-Binomial null model and the reference model where the links are established separately for each vote type, see left-hand side of Fig. 6. For the inference of a political network, we see a significant overlap between the links inferred via the two approaches, evidenced by a high Jaccard index. Nevertheless, we observe a set of method-unique links. The reference model yields from around a hundred to almost a thousand unique links depending on the statistical significance level in link validation. Our proposed approach yields between 100 and less than ten unique links depending on the statistical threshold.

### Investor network

The shareholding data used in this analysis comes from the Central Finnish Depository Register provided by Euroclear Finland. Our sample data consist of all marketplace transactions executed over $N=253$ trading days from 1 January 2008 to 31 December 2008 in the Helsinki Stock Exchange by *Nokia stock* shareholders. This includes more than 48 thousand investors. Households are the most abundant investor group in the stock market, yet the majority of them are rather passive investors making infrequent trades in a few securities^60,61^. As there needs to be a sufficient number of observations to establish statistically strong links for the passive investors, we focus the analysis on the active investors. We consider an investor as active if she traded on at least ten trading days during the calendar year. This reduces the number of investors from 48 to 3.1 thousand. The selected set of investors is composed of: households (84.81%), non-financial corporations (10.31%), financial-insurance corporations (2.33%), non-profit institutions (0.83%), government institutions (0.67 %), EU institutions (0.67%), non-EU institutions (0.29%), and foreign investors (0.1%). A more detailed description of the data set can be found in a number of recently published papers^45,62–65,67^.

First, for each investor (agent) *i* and each trading day (event) *t*, we calculate the net-scaled volume as

7 $$\begin{aligned} v_{i,t} = \frac{B_{i,t}-S_{i,t}}{B_{i,t}+S_{i,t}}, \end{aligned}$$

where $B_{i,t}$ and $S_{i,t}$, respectively, are the total purchased and sold Nokia stock volumes by investor *i* on day *t*^25,40,66^. Based on the net-scaled volumes and an arbitrarily chosen threshold $\theta = 0.1$ we assign one of three trading states – *b* (primarily buying, when $v_{i,t} > \theta$), *s* (primarily selling, when $v_{i,t} < -\theta$), or *bs* (day trading, when $-\theta< v_{i,t} < \theta$). In this case, the set of possible link types is defined as ${\textbf{a}} = \{b, s, bs\}$.

**Figure 7:**
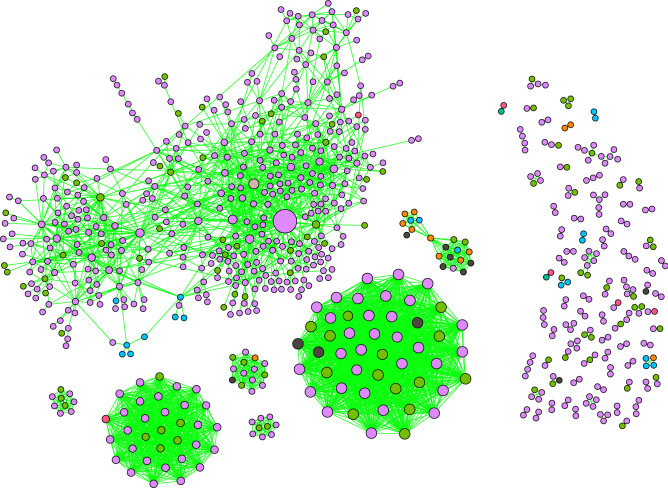
Validated investor network inferred from bipartite data where investors trade Nokia stock. Node colors indicate the sectors of investors. —households, —non-financial corporations, —financial and insurance corporations, —non-profit institutions, —government institutions, —EU institutions, —non-EU institutions, —foreign investors.

Next, we count the trading days when an investor was active $N_i$. We also estimate the empirical probabilities of choosing different trading actions – $p_i(a_k)$, where $a_k \in \{b, s, bs\}$. Finally, for all pairs of investors (*i*, *j*), we calculate the number of trading days when they both had the same (${\tilde{N}}_{ij}$) and opposing ($N_{ij}^d$) trading states. Moreover, we calculate the probabilities of choosing the same and opposing trading states by chance $p_{ij}$ and $q_{ij}$. Here, we consider the day-trading state *bs* as a neutral state. Naturally, the buying state *b* is the opposite of the selling state *s*. With these parameters, we calculate the *p* values that represent how likely it is to observe similar or more extreme values than ${\tilde{N}}_{ij}$ and $N_{ij}^d$ by chance. We pass the resulting *p* values through the Bonferroni multiple test correction with the unadjusted statistical threshold $\alpha = 0.01$. The resulting investor network is presented in Fig. 7.

Overall, our proposed validation method finds 3236 links among 794 investors. The network consists of 89 connected components, where the largest component contains 56 % of the nodes. The following six biggest components form almost completely connected subgraphs. The network contains 3233 positive and only three negative links. When investigating the triadic motifs in the signed investor network, we find that all of them are stable.

Finally, on the right-hand side of Fig. 6, we compare the inferred investor network against the network inferred using the reference null model. We find that the networks inferred by the two approaches are significantly different. The Jaccard index that measures the overlap between the sets of inferred links varies between just above 0.6 to just above 0.3 when the statistical validation threshold is less than 0.1.

## Discussion and conclusions

Collecting data about direct relationships between agents is only sometimes feasible. For this reason, statistical tools are needed to extract such information from the rich data sets that exist in the form of bipartite networks. Indeed, many data sets generated by human behavior can be represented as bipartite networks: one set of nodes represents the individuals, and the other represents events.

In this paper, we propose a novel approach to validate projections of bipartite networks. Our approach is tailored for data sets with a bipartite structure having links of qualitatively different types. State-of-the-art methods perform the projection for each link type separately, yielding a network for each link type or completely ignoring the differences. Unlike other null models, our proposed model integrates information about all link types, yielding a single network. Our method considers the heterogeneity of agent activity levels and their preferences for different link types. The link validation procedure does not require an arbitrary threshold but rather a level of statistical significance to differentiate between strong and weak associations. Moreover, the introduced link validation is ideal for inferring signed networks when agents’ links in the bipartite network can be considered positive or negative.

With synthetic data, we show the performance differences of our method against the state-of-the-art. We identified that our method underperforms only in extreme situations in the network reconstruction exercise. In particular, our method produces more false positives when the agents are highly active and likely to copy neighbors’ behavior. However, the effects can be moderated by applying multiple test corrections.

We showed that our method could capture different topologies with investment and political data sets. In the future, we will research the possibility of using the *p* values resulting from our link validation as an early warning signal for forthcoming structural changes in the system.

## Supplementary Information

Supplementary Information.

## Data Availability

The data that support the findings of this study are available from Euroclear Finland Ltd.; however, the raw data cannot be distributed by the authors under the non-disclosure agreement signed with the data provider. The data used to infer political networks is publicly available from the official website of the parliament www.lrs.lt or by request from the authors.
