# Supplementary material for: Inference of monopartite networks from bipartite systems with different link types
Source: Sci Rep. 2023 Jan 19;13:1072. doi: 10.1038/s41598-023-27744-8 (PMC9852298; doi:10.1038/s41598-023-27744-8)
Supplement: Supplementary file 1 — Supplementary Information. [file 41598_2023_27744_MOESM1_ESM.pdf]

Inference of monopartite networks from bipartite systems with different link types

Kęstutis Baltakys[1,*]

[1]Statistical Data Analytics, Faculty of Information Technology and Communication Sciences, Tampere University, Finland
[*]kestutis.baltakys@tuni.fi

ABSTRACT

Many of the real-world data sets can be portrayed as bipartite networks. Since connections between nodes of the same type are lacking, they need to be inferred. The standard way to do this is by converting the bipartite networks to their monopartite projection. However, this simple approach renders an incomplete representation of all the information in the original network. To this end, we propose a new statistical method to identify the most critical links in the bipartite network projection. Our method takes into account the heterogeneity of node connections. Moreover, it can handle situations where links of different types are present. We compare our method against the state-of-the-art and illustrate the findings with synthetic data and empirical examples of investor and political data.

Supplementary Information

A Improvement in single link detection

Our proposed method takes into account overlap in all link types. Looking at the minimum p-value obtained with the Hypergeometric null model over all link types does not consider overlaps in other link types. In order to have a better comparison, we average p-values or take the maximum observed over all link types. We illustrate the difference in p-values of the Hypergeometric-Binomial and the alternative reference models in Fig. S1.

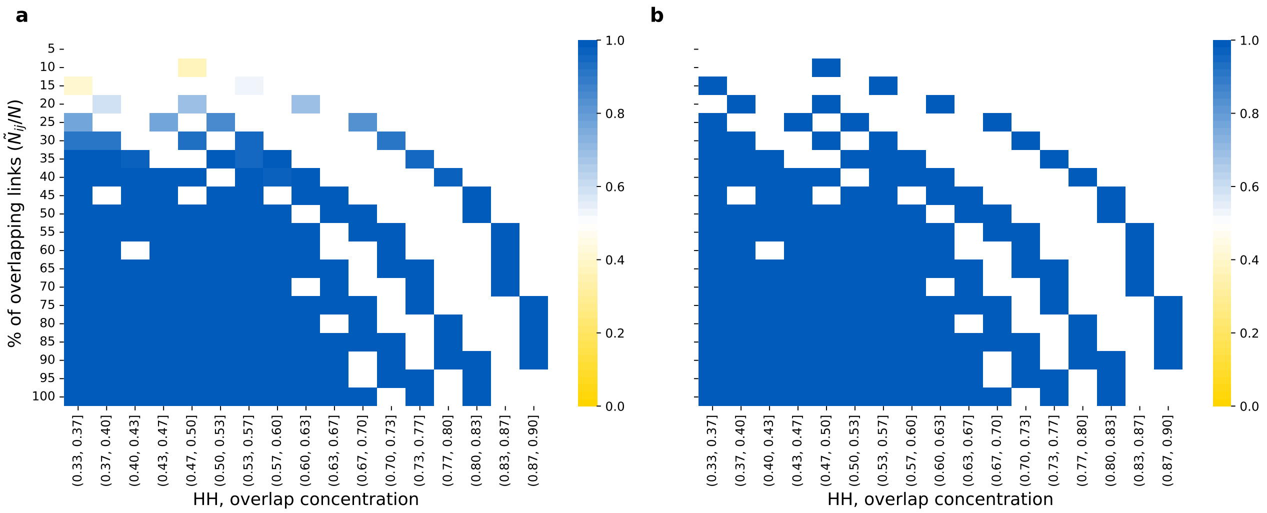

Figure S1. Figures **a** and **b** share the same axes. The horizontal axis shows the concentration of overlap in terms of link types measured with the Herfindahl–Hirschman index, see Eq. 6. The vertical axis indicates the fraction of events to which both agents are connected via the same link types ($\tilde{N}_{ij}/N$). Figure **a** depicts the fraction of cases where our proposed model yields a lower p-value than the mean p-value obtained using the Hypergeometric case. Similarly, Fig. **b** depicts the fraction of cases where our proposed model yields a lower p-value than the max p-value obtained using the Hypergeometric case.

B Influence model

During the synthetic bipartite network generation, events are added one by one, and nodes decide if and how to connect to them. First, for a given event, a random node in the social network decides the link type to connect to that event according to its link type preferences. Next, the procedure goes as follows:

(a) select the neighbors of nodes that have decided about the connection to the event
(b) in a random sequence one by one selected neighbors from the step (a) and do steps (c) – (e)
(c) the selected node i decides whether it will connect to the current event with probability n_i
(d) if the node decides to connect to the event, next with probability c_i it decides whether it will mimic one of its neighbors
(e) if the node decides to mimic a neighbor, it randomly selects one of the link types that the neighbors have used, otherwise it randomly chooses which link type to use according to its link preference attribute
(f) if there are still nodes that have not decided about the connection to current event return to step (a).

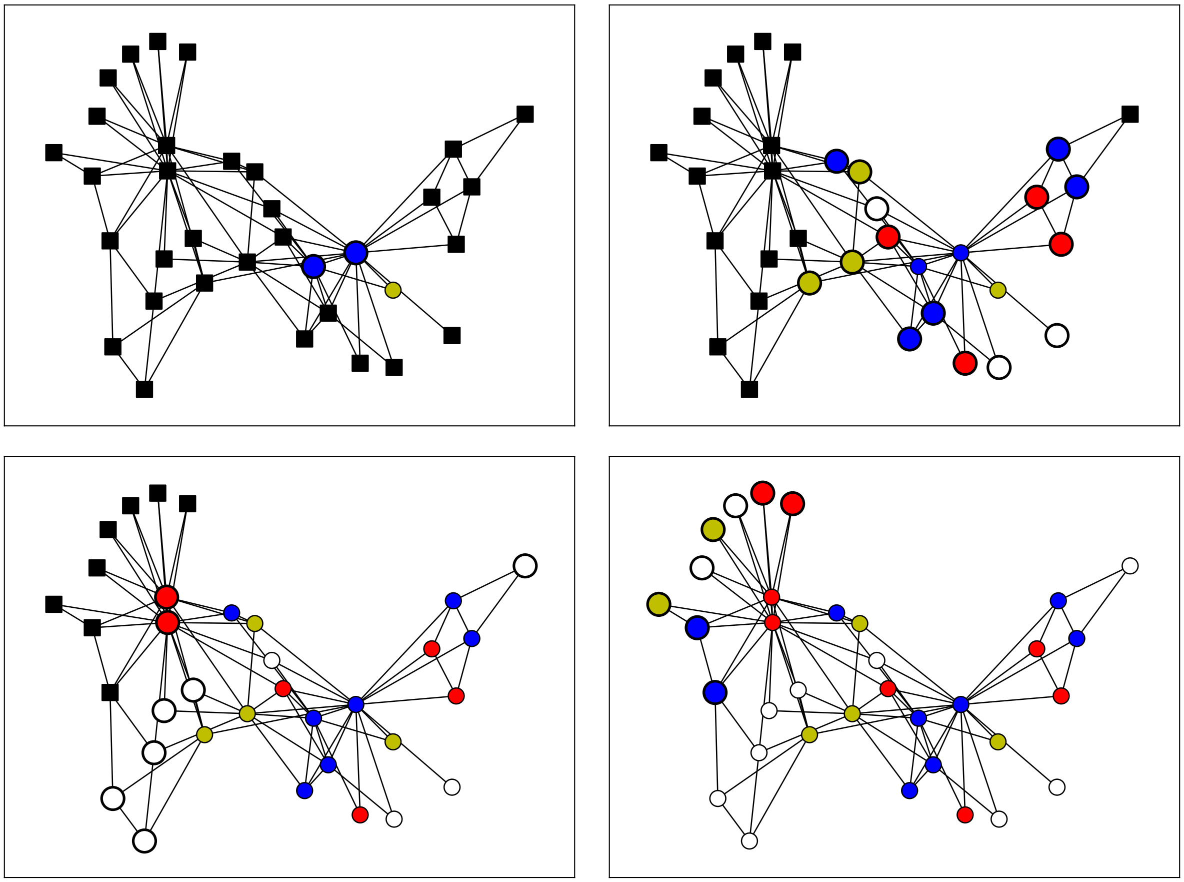

Figure S2. An illustration of the influence model used to generate synthetic bipartite networks over a ground truth social network.

Fig. S2 illustrates how the synthetic data over a ground truth social network is generated. Nodes in the form of a black square represent agents that have not yet decided on the connection to the event. Nodes in the form of a white circle represent agents that have decided not to connect to the event. Colored circles represent nodes that have decided to connect to the network via one of the three link types. Link types are represented in yellow, blue, and red. Smaller circles indicate the nodes that have decided on the connection in the previous iteration. Larger nodes are the ones deciding the currently illustrated iteration.

The top left network illustrates the first two iterations of the procedure. First, a random node decides to connect to the event via a yellow link. Then the two neighbors decide to connect to the event via a blue link type. One of the nodes that chose the blue connection did that independently. The other node observed the neighbor's choice of blue connection. It could have chosen the blue connection either by mimicking its neighbor's behavior or independently deciding on it. The remaining three figures illustrate the further spread of decisions about the connection types.

C Evaluation of ground-truth network reconstruction

Validated networks are evaluated against ground-truth network with four performance measures defined in Table S1.

Measure	Accuracy	Precision	Recall	F1
Formula	$\frac{tp+tn}{tp+tn+fp+fn}$	$\frac{tp}{tp+fp}$	$\frac{tp}{tp+fn}$	$2\cdot\frac{\text{Precision}\cdot\text{Recall}}{\text{Precision}+\text{Recall}}$

Table S1. Performance measures used to evaluate validated networks. The count of true positives is denoted as *tp*, true negatives – *tn*, false positives – *fp*, and false negatives – *fn*.

In Table S2, we present the sensitivity of network reconstruction to the three critical parameters of the influence model. In particular, Panel A presents the sensitivity of network reconstruction when we fix the agent link preference to specific values resulting in different probabilities of random link overlaps p_{ij}. Panels B and C present the sensitivity of the ground-truth network reconstruction when node activity n_i and coping parameters c_i are fixed, respectively.

Table S2. Sensitivity of network reconstruction performance to influence model parameters. All networks are validated with statistical significance of $\alpha = 0.001$.

Panel A: Sensitivity to link preferences, p_{ij}

		precision			recall			F1			accuracy			AUC	
p_{ij}	⟨pr⟩	⟨Δpr⟩	≥	⟨re⟩	⟨Δre⟩	≥	⟨F1⟩	⟨ΔF1⟩	≥	⟨acc⟩	⟨Δacc⟩	≥	⟨AUC⟩	⟨ΔAUC⟩	≥
0.33	82.8%	0.3%	51%	20.6%	7.6%***	100%	32.6%	10.4%***	100%	88.3%	0.8%***	99%	77.9%	2.4%***	100%
0.47	86.3%	4.3%***	86%	16.1%	3.8%***	100%	27.0%	5.8%***	100%	88.0%	0.5%***	100%	76.8%	1.9%***	100%
0.60	88.5%	6.2%***	92%	12.1%	1.5%***	93%	21.2%	2.4%***	93%	87.6%	0.3%***	94%	75.6%	1.3%***	98%
0.73	91.0%	8.1%***	95%	9.5%	−0.1%	50%	17.2%	0.1%	50%	87.3%	0.1%***	82%	75.1%	0.7%**	86%
0.87	90.9%	5.9%***	93%	7.5%	−0.5%*	30%	13.9%	−0.8%*	30%	87.0%	0.0%	65%	73.8%	−0.1%	43%
1.00	92.0%	0.0%	100%	6.5%	0.0%	100%	12.0%	0.0%	100%	86.9%	0.0%	100%	72.3%	0.0%	94%

Panel B: Sensitivity to activity parameter, n_i

n_i	⟨pr⟩	⟨Δpr⟩	≥	⟨re⟩	⟨Δre⟩	≥	⟨F1⟩	⟨ΔF1⟩	≥	⟨acc⟩	⟨Δacc⟩	≥	⟨AUC⟩	⟨ΔAUC⟩	≥
(0.0, 0.1)	80.7%	1.1%	98%	0.1%	0.0%	96%	2.7%	−0.1%	91%	86.1%	0.0%*	96%	55.7%	−0.0%	43%
(0.1, 0.2)	93.3%	6.6%***	95%	1.1%	0.4%***	87%	4.0%	0.7%***	86%	86.2%	0.1%***	87%	68.4%	0.0%	52%
(0.2, 0.3)	94.0%	6.9%***	89%	3.6%	1.2%***	82%	7.9%	2.2%***	81%	86.6%	0.2%***	82%	75.8%	0.7%***	73%
(0.3, 0.4)	94.2%	5.8%***	83%	8.5%	2.7%***	88%	15.5%	4.5%***	87%	87.2%	0.4%***	89%	79.5%	1.5%***	85%
(0.4, 0.5)	91.8%	4.8%***	74%	14.9%	3.9%***	89%	25.2%	6.0%***	88%	88.0%	0.6%***	89%	82.1%	2.1%***	92%
(0.5, 0.6)	86.9%	2.7%***	64%	23.4%	5.0%***	92%	36.0%	6.4%***	89%	88.8%	0.7%***	88%	83.5%	2.0%***	92%
(0.6, 0.7)	80.4%	0.8%*	55%	30.4%	4.5%***	87%	42.7%	4.7%***	83%	89.1%	0.4%***	77%	83.9%	1.6%***	87%
(0.7, 0.8)	72.3%	−0.7%	45%	36.7%	3.2%***	81%	46.8%	2.2%***	72%	88.8%	0.1%	60%	83.9%	1.2%***	81%
(0.8, 0.9)	65.2%	−0.2%	45%	40.7%	0.9%*	64%	47.9%	0.1%	53%	88.0%	−0.2%*	50%	83.1%	0.7%***	69%
(0.9, 1.0)	57.8%	0.5%	50%	42.4%	−2.7%***	30%	46.3%	−2.1%***	28%	86.6%	−0.2%*	46%	81.7%	0.1%	53%

Panel C: Sensitivity to copying parameter, c_i

c_i	⟨pr⟩	⟨Δpr⟩	≥	⟨re⟩	⟨Δre⟩	≥	⟨F1⟩	⟨ΔF1⟩	≥	⟨acc⟩	⟨Δacc⟩	≥	⟨AUC⟩	⟨ΔAUC⟩	≥
(0.0, 0.1)	23.3%	1.7%	87%	0.0%	−0.1%***	94%	2.5%	0.0%*	100%	86.1%	0.0%***	94%	54.9%	0.1%	52%
(0.1, 0.2)	54.9%	4.9%	88%	0.1%	−0.3%***	76%	2.5%	−0.3%*	84%	86.1%	−0.0%*	78%	63.6%	0.6%**	64%
(0.2, 0.3)	91.1%	9.9%***	90%	0.6%	−0.7%***	52%	3.3%	−1.3%***	51%	86.2%	−0.1%***	57%	70.6%	1.4%***	81%
(0.3, 0.4)	95.2%	8.6%***	93%	2.5%	−1.1%***	42%	6.0%	−1.8%***	44%	86.4%	−0.1%***	53%	76.0%	2.0%***	91%
(0.4, 0.5)	96.7%	8.9%***	90%	7.8%	0.0%	59%	14.4%	0.1%	57%	87.1%	0.1%***	66%	80.0%	2.4%***	95%
(0.5, 0.6)	94.2%	6.9%***	85%	16.8%	3.2%***	85%	27.9%	4.9%***	84%	88.3%	0.6%***	89%	82.7%	2.6%***	99%
(0.6, 0.7)	86.9%	5.4%***	75%	30.0%	8.0%***	98%	43.2%	9.6%***	97%	89.5%	1.1%***	96%	84.5%	2.6%***	99%
(0.7, 0.8)	72.9%	0.8%	52%	43.9%	11.7%***	99%	52.7%	9.8%***	98%	89.4%	0.9%***	82%	84.7%	2.5%***	100%
(0.8, 0.9)	55.2%	−4.2%***	22%	55.7%	13.3%***	100%	52.9%	5.8%***	84%	86.2%	−0.9%***	40%	84.3%	2.1%***	98%
(0.9, 1.0)	39.4%	−5.3%***	7%	66.8%	13.2%***	100%	47.7%	1.1%***	54%	78.8%	−4.0%***	7%	82.7%	1.6%***	95%

*** $p < 0.001$; ** $p < 0.01$; * $p < 0.1$

Table S3 compares the ground-truth network reconstruction against alternative reference models. In particular, Panel A compares to the reference model where the mean p-value resulting from the Hypergeometric null is used in link validation. Similarly, Panel B shows the comparison where the maximum p-value is used in the reference model.

An alternative way to measure the performance of ground-truth network reconstruction, is to investigate how often individual links in the ground-truth network are (miss-)identified. We simulate 100 bipartite networks using the same influence model as before. We illustrate the performance of the reference method for all three link types (Fig. S3A-C). Fig. S3D illustrates the performance of our method. Here, we use the statistical significance of 0.01 adjusted by the number of tests performed (Bonferroni multiple test correction). We perform 561 tests for each network using our method and the reference method

Table S3. The relationship between the network reconstruction performance measures and statistical link validation threshold α for differently defined reference models. The columns with $\langle\cdot\rangle$ indicate the averaged measures obtained with our method. The columns with $\langle\Delta\cdot\rangle$ indicate the mean difference between the measures obtained with our and reference null models. Here the stars indicate the p-values for a paired two-sample mean t-test, with an alternative that the mean difference of the samples is not zero. Finally, the $\geq$ columns indicate the fraction of simulations where the measures obtained with our null model yielded a similar or better performance compared to the reference model. All results are obtained over 1000 simulations.

Panel A: comparison to the reference model where the *mean* p-value over the three link types is used in network validation

		precision			recall			F1			accuracy	
α	$\langle$pr$\rangle$	$\langle\Delta$pr$\rangle$	$\geq$	$\langle$re$\rangle$	$\langle\Delta$re$\rangle$	$\geq$	$\langle$F1$\rangle$	$\langle\Delta$F1$\rangle$	$\geq$	$\langle$acc$\rangle$	$\langle\Delta$acc$\rangle$	$\geq$
10^{-10}	85.0%	$-15.0\%^{*}$	55%	1.3%	$1.3\%^{***}$	100%	9.2%	$6.7\%^{**}$	100%	86.3%	$0.2\%^{***}$	100%
10^{-9}	89.7%	$-10.3\%^{**}$	62%	1.7%	$1.7\%^{***}$	100%	8.8%	$6.2\%^{***}$	100%	86.3%	$0.2\%^{***}$	100%
10^{-8}	90.6%	$-9.4\%^{**}$	60%	2.4%	$2.4\%^{***}$	100%	11.9%	$9.1\%^{***}$	100%	86.4%	$0.3\%^{***}$	100%
10^{-7}	90.1%	$-9.9\%^{***}$	56%	3.4%	$3.3\%^{***}$	100%	12.9%	$9.9\%^{***}$	100%	86.5%	$0.4\%^{***}$	100%
10^{-6}	87.5%	$-11.0\%^{***}$	40%	4.9%	$4.7\%^{***}$	100%	15.5%	$12.4\%^{***}$	100%	86.7%	$0.6\%^{***}$	100%
10^{-5}	86.6%	$-12.5\%^{***}$	34%	7.1%	$6.8\%^{***}$	100%	18.6%	$15.5\%^{***}$	100%	87.0%	$0.9\%^{***}$	100%
10^{-4}	86.0%	$-13.4\%^{***}$	28%	10.7%	$10.0\%^{***}$	100%	23.2%	$19.4\%^{***}$	100%	87.4%	$1.2\%^{***}$	100%
10^{-3}	82.9%	$-15.0\%^{***}$	15%	16.7%	$15.1\%^{***}$	100%	30.2%	$25.0\%^{***}$	100%	87.9%	$1.6\%^{***}$	99%
10^{-2}	72.6%	$-23.2\%^{***}$	4%	27.8%	$23.2\%^{***}$	100%	40.1%	$30.5\%^{***}$	100%	88.3%	$1.6\%^{***}$	94%
10^{-1}	46.5%	$-35.4\%^{***}$	0%	50.6%	$34.2\%^{***}$	100%	47.6%	$21.3\%^{***}$	98%	84.6%	$-3.1\%^{***}$	10%

Panel B: comparison to the reference model where the *max* p-value over the three link types is used in network validation

10^{-10}	76.5%	$-23.5\%^{**}$	29%	1.3%	$1.3\%^{***}$	100%	9.3%	$6.8\%^{*}$	100%	86.3%	$0.2\%^{***}$	100%
10^{-9}	87.3%	$-12.7\%^{**}$	54%	1.7%	$1.7\%^{***}$	100%	9.9%	$7.2\%^{***}$	100%	86.3%	$0.2\%^{***}$	100%
10^{-8}	89.9%	$-10.1\%^{**}$	59%	2.4%	$2.4\%^{***}$	100%	11.9%	$9.1\%^{***}$	100%	86.4%	$0.3\%^{***}$	100%
10^{-7}	89.3%	$-10.7\%^{***}$	54%	3.4%	$3.3\%^{***}$	100%	13.0%	$10.0\%^{***}$	100%	86.5%	$0.4\%^{***}$	100%
10^{-6}	86.5%	$-13.3\%^{***}$	37%	4.9%	$4.8\%^{***}$	100%	15.8%	$12.9\%^{***}$	100%	86.7%	$0.6\%^{***}$	100%
10^{-5}	85.8%	$-12.7\%^{***}$	33%	7.1%	$6.9\%^{***}$	100%	18.4%	$15.2\%^{***}$	100%	87.0%	$0.9\%^{***}$	100%
10^{-4}	84.7%	$-14.6\%^{***}$	24%	10.7%	$10.2\%^{***}$	100%	23.4%	$19.9\%^{***}$	100%	87.4%	$1.2\%^{***}$	100%
10^{-3}	81.6%	$-17.6\%^{***}$	10%	16.7%	$15.6\%^{***}$	100%	31.0%	$26.4\%^{***}$	100%	87.9%	$1.7\%^{***}$	99%
10^{-2}	71.6%	$-25.5\%^{***}$	3%	27.8%	$24.6\%^{***}$	100%	40.5%	$33.0\%^{***}$	100%	88.3%	$1.8\%^{***}$	95%
10^{-1}	46.5%	$-41.2\%^{***}$	0%	50.6%	$40.3\%^{***}$	100%	47.6%	$29.7\%^{***}$	99%	84.6%	$-2.6\%^{***}$	15%

*** $p < 0.001$; ** $p < 0.01$; * $p < 0.1$

when applied to each link type separately. Note that we perform three times more tests when taking the minimum value of the reference method over the three link types. Therefore the multiple test correction is for 1683 tests in total. The intensity of green lines indicates how often the ground truth network links are correctly identified. Our method is more likely to infer true links correctly (see the histogram of intensities in Fig. S5). The intensity of red lines specifies how often different methods infer links that do not exist in the ground truth network. The complementary cumulative distributions in Fig. S3E and F report the probability that the different methods would have more than a certain number of true or false positives. Our method is more likely to produce a significantly higher number of true positives and only a marginally higher number of false positives. Without the multiple test correction, our method is on par with the best alternative in terms of true positives, see Fig. S4. However, our method is much more likely to have fewer false positives.

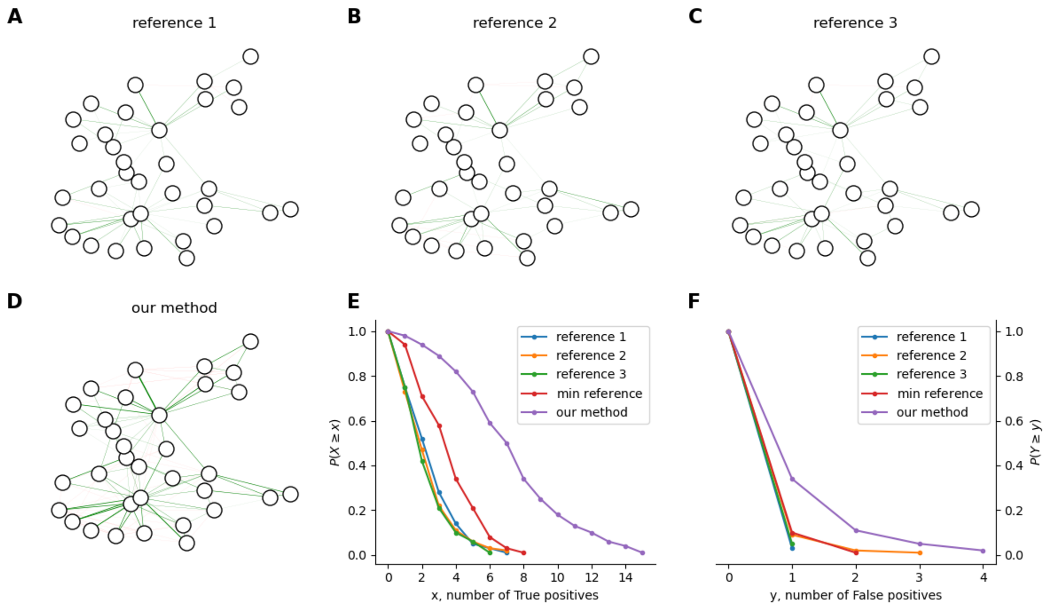

Figure S3. Comparison of network reconstruction performance when Bonferroni multiple test correction is applied. A-C illustrate the average reconstructed network using the reference method. D illustrates the average reconstructed method using our method. The intensity of green (red) links indicates how often the true (false) link is inferred. E and F illustrate the complementary cumulative distributions for the number of true and false positives, respectively.

Fig. S5 illustrates the probability for a true link to be correctly identified by our and reference method applied to each link type individually and altogether. The observations come from the experiment illustrated in article.

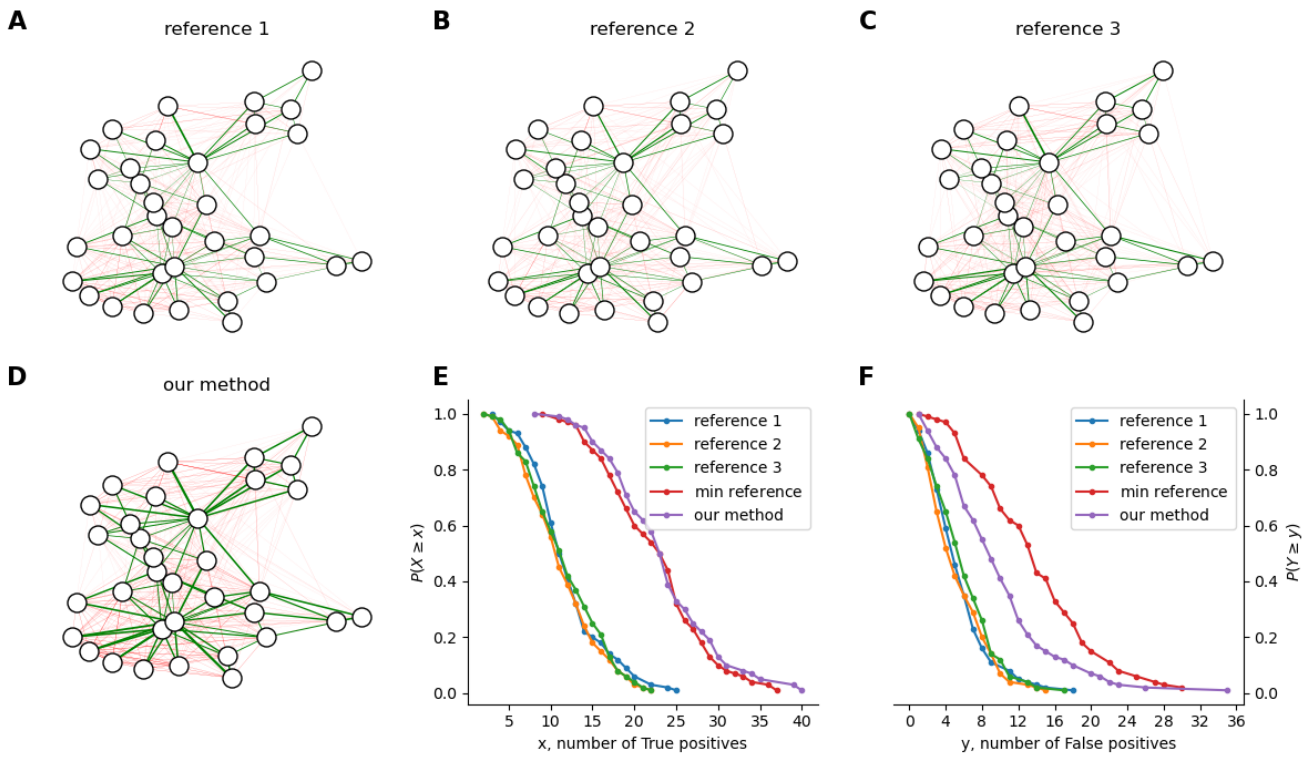

Figure S4. Comparison of network reconstruction performance when no multiple test correction is applied. A-C illustrate the average reconstructed network using the reference method. D illustrates the average reconstructed method using our method. The intensity of green (red) links indicates how often the true (false) link is inferred. E and F illustrate the complementary cumulative distributions for the number of true and false positives, respectively.

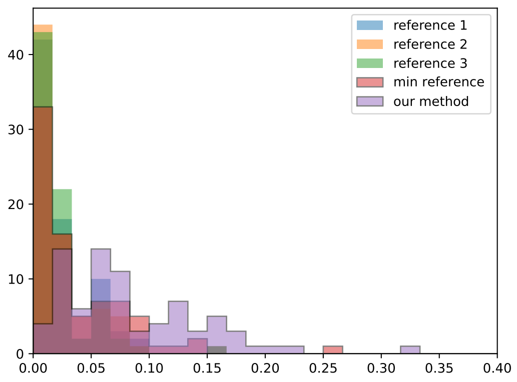

Figure S5. Probabilities to correctly infer true links with multiple test correciton.

D Probability mass function of the hypergeometric-binomial mixture distribution

The probability mass function of the hypergeometric-binomial mixture distribution, see Fig. S6, is defined as

$$\begin{aligned}\mathbb{P}(Y = N_{ij}^s | N, N_i, N_j, p_{ij}) &= \sum_{X=N_{ij}^s}^{\min(N_i,N_j)} H(X|N,N_i,N_j) B(Y = N_{ij}^s | X, p_{ij}) \\ &= \sum_{X=N_{ij}^s}^{\min(N_i,N_j)} \frac{\binom{N_i}{X}\binom{N-N_i}{N_j-X}}{\binom{N}{N_j}} \binom{X}{N_{ij}^s} p_{ij}^{N_{ij}^s} (1-p_{ij})^{X-N_{ij}^s}.\end{aligned} \tag{1}$$

Here, the hypergeometric distribution $H(X|N,N_i,N_j)$ part determines the probability that agent's i and j would both choose the same X events to take any action given that agent i (j) has taken action on N_i (N_j) events out of N possible occasions. The binomial component determines the probability that on Y of those X events, they would choose, e.g., the same action, whatever it is, with probability p_{ij}. Here the probability to take the same action p_{ij}, can be determined either by observing the likelihood to choose a certain action over the whole population, in which case p_{ij} would be identical for all pairs of agents, or it can be pair-specific, in which case it would take into account how likely both agent's are to choose different actions.

If both agents take actions on all possible occasions, in which case $N_i = N_j = N$, then the probability of observing agents taking the same action Y times reduces to

$$\mathbb{P}(Y = N_{ij}^s | N, N_i = N, N_j = N, p_{ij}) = B(Y|N, p_{ij}).$$

Suppose there is only one type of action that both agents can choose from, or they would always choose the same action among the possible alternatives, i.e., $p_{ij} = 1$. In that case, the probability of observing Y events when agents i and j take the same actions reduces to the hypergeometric probability mass function

$$\mathbb{P}(N_{ij}^s = Y | N, N_i, N_j, p_{ij} = 1) = H(N_{ij} = Y | N, N_i, N_j).$$

When validating the synchronization between agents' actions, given the number of days they are active and their preferences for different actions, we test the null hypothesis that agents randomly select the days to act on and actions to take. The corresponding p-value of the null hypothesis is defined as

$$\mathbb{P}(Y \geq N_{ij}^s | N, N_i, N_j, p_c) = \sum_{X=N_{ij}^s}^{\min(N_i,N_j)} \sum_{Y=N_{ij}^s}^{X} H(X|N,N_i,N_j) B(Y = N_{ij}^s | X, p_{ij}). \tag{2}$$

Suppose we observe a sufficiently small p-value and reject the null hypothesis that the observed synchronization came about by chance. In that case, we can establish a link between the i and j in the synchronization network.

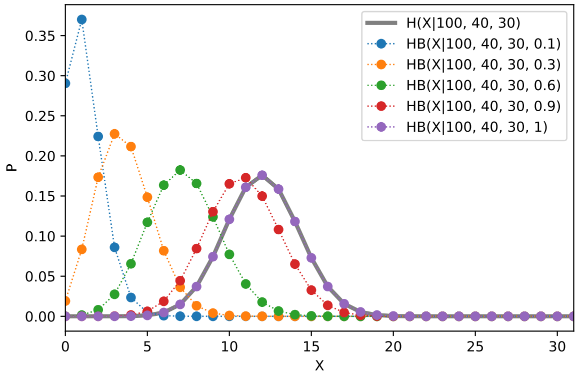

Figure S6. Probability mass function $\mathbb{P}(N_{ij}^s = X | N = 100, N_i = 40, N_j = 30, p_{ij})$ with $N = 100$, $N_i = 40$, $N_j = 30$, for different probabilities to choose the same action $p_{ij} \in (0.1, 0.3, 0.6, 0.9, 1)$ as defined in Eq. 1.
